# Diagnostic delay in children with inflammatory bowel disease in the German-Austrian patient registry CEDATA-GPGE 2014–2018

**DOI:** 10.1038/s41598-022-25487-6

**Published:** 2022-12-07

**Authors:** Maren Leiz, Melanie Knorr, Kilson Moon, Luisa Tischler, Jan de Laffolie, Neeltje van den Berg

**Affiliations:** 1 Institute for Community Medicine, University Medicine, Greifswald, Germany; 2 General Pediatrics & Neonatology, Justus-Liebig-University, Giessen, Germany

**Keywords:** Inflammatory bowel disease, Paediatrics

## Abstract

The incidence and prevalence of pediatric-onset inflammatory bowel disease (PIBD) are on the rise worldwide. Initial symptoms are often recognized with a delay, which reduces the quality of life and may lead to an increased rate of complications. The aim of this study was to determine the diagnostic delay in PIBD and to identify potential influencing factors. Therefore, data from the German-Austrian patient registry CEDATA-GPGE for children and adolescents with PIBD were analyzed for the period January 2014 to December 2018. There were 456 children identified in the data, thereof 258 children (57%) with Crohn’s disease (CD) and 198 children (43%) with Ulcerative colitis (UC). The median age was 13.3 years (interquartile range (IQR) = 10.9−15.0), and 44% were females. The median diagnostic delay was 4.1 months (IQR = 2.1–7.0) in CD and 2.4 months (IQR = 1.2–5.1) in UC (p = 0.01). UC was associated with earlier diagnosis than CD (p < 0.001). Only a few factors influencing the diagnostic delay have been verified, e.g., abdominal pain at night and if video capsule endoscopy was performed. Diagnostic delay improved over the years in participating centers, but the level of awareness needs to be high even in common symptoms like abdominal pain.

## Introduction

Inflammatory bowel diseases (IBD) include Crohn’s disease (CD), Ulcerative colitis (UC), and unclassified inflammatory bowel disease (IBD-U). Approximately 20% of the patients are diagnosed in the first two decades of life^1^. Incidence and prevalence are on the rise worldwide with a steep increase in pediatric-onset IBD (PIBD)^2–4^. Between the years 2010 and 2020, 84% of all PIBD studies worldwide reported an increase in incidence and all studies reported an increasing prevalence^4^.

Germany is amongst the group of highest incidence countries worldwide, like Canada, the UK, and the US^5^. PIBD incidence in Germany is estimated to be 17.41/100,000 children in 2012 (CD 10.6; UC 6.15) from health insurance data^5^. PIBD can significantly impair the development of children and adolescents, e.g. pubertal development, growth, social and psychological development, and education^6,7^.

It can be challenging to differentiate PIBD from a large variety of diseases and conditions, such as functional gastrointestinal diseases, infection, eating disorders, malnutrition, malignancy, or extraintestinal manifestation mimicking skin, liver, joint, or bone disease. Initial symptoms (e.g., abdominal pain, growth delay, diarrhea) may be interpreted differently, which leads to diagnostic delay, reduces quality of life, and may lead to more complications^1,8,9^.

Since 2004, German and Austrian pediatric gastroenterologists can document diagnostic and treatment data of children and adolescents with PIBD in the patient registry CEDATA-GPGE. The aim of this registry is to obtain data on epidemiology, patterns of involvement, diagnosis, treatment, and quality of care of children and adolescents with PIBD^1^.

Studies on the development and influencing factors of diagnostic delay are essential given the obvious importance of the issue and the potential to reduce delay and therewith reduce impairment of patients’ lives, cost of care, complications, and e.g., final adult height in CD^9,10^. For UC, a longer time to diagnosis was associated with one of the most important prognostic factors, namely a higher rate of more extensive inflammation^11^. In the last decade many factors in German Health Care and caring for pediatric IBD patients in general have changed (e.g., new therapies, new phenotypes, rising incidence), that follow-up analyses are relevant.

The aim of this study was to analyze the diagnostic delay in children and adolescents with IBD, i.e. the time between first symptoms and the confirmed diagnosis of IBD, and to identify influencing factors on the basis of the patient registry CEDATA-GPGE in Germany and Austria.

## Methods

The analyses were based on data from the CEDATA-GPGE registry. This registry has been founded in 2004 by the association of pediatric gastroenterology and nutrition (Gesellschaft für Pädiatrische Gastroenterologie und Ernährung GPGE e.V.). It collects clinical and paraclinical data of children and adolescents with IBD in German-speaking countries, currently Germany and Austria. Participation and documentation in the registry are voluntary and mainly carried out by certified pediatric gastroenterology centers. The data collected include initial presentation, history, signs and symptoms, laboratory, endoscopy and radiology results, initial therapy and response to therapy as well as follow-up. The initial period is defined as the first three months, follow-up is recommended at every patient visit, but at least twice a year. The registry contains data of more than 6,000 children and adolescents and includes over 50,000 documentations of patient contacts^1^.

We analyzed the initial documentation (first three documented months, see Additional File 1) of children and adolescents with a first diagnosis of CD or UC between January 2014 and December 2018, whose documentation was available in the registry no later than 3 months after diagnosis. Children and adolescents with unclassified IBD were excluded from this analysis. Diagnostic delay was determined as the median time in months between the date of first symptoms and the date of diagnosis. As first symptoms, we defined self-reported first symptoms by the children and for younger children reported by their caregivers. Potential factors influencing diagnostic delay were identified using univariate Cox regression, including demographics, presenting symptoms, disease phenotype, diagnostic procedures, and other factors. Therefore, we took the appropriate diagnostic measures from the Porto criteria^12^. The potential factors were examined with the proportional hazards model and presented as hazard ratios (HR) with 95% confidence intervals. HR < 1.0 represent factors associated with late diagnosis. A Chi-square test was used for categorical variables and a Kruskal–Wallis rank sum test was used for continuous variables. The significance level was *P* < 0.05.

Dichotomized variables of age were chosen since, in clinical reasoning, age is not a continuous variable, especially in IBD, but is structured in age groups with different disease behavior. Gastroenterological centers were categorized on the basis of the number of pediatric IBD patients per year as small (< 25 patients), medium (25–100 patients), and large (> 100 patients), as reported in the quality reports of the Federal Joint Committee in 2016. The Paris classification was used for disease location (L) in CD (ileal disease = L1 or L1 + L4) and for disease extent (E) in UC^13^. Variables included like extraintestinal manifestation (EIM) or perianal disease are defined in the registry dataset. For EIM the definition includes any extraintestinal manifestation suspected by the treating specialist and is further structured in arthritis (peripheral, axial), hepatobiliary involvement, and skin among others. Perianal disease refers to any anal finding beyond erythema or small tags. Abdominal findings are a variable that includes any findings during the physical exam of the abdomen (pain, tenderness, resistance, etc.).

Data processing and statistical calculations were performed with SAS Enterprise Guide 7.1 (SAS Institute Inc, Cary, North Carolina). Analyses on the basis of the registry CEDATA-GPGE were approved by the Ethics Committee of the Justus-Liebig University Giessen (ethics approval protocol number 07/11) and by all ethics committees of the centers involved. Participating centers from Austria have an additional local ethics vote. The analyses were performed in accordance with the guidelines and recommendations for Good Epidemiological Practice^14^ and in accordance with the Declaration of Helsinki^15^. The parents of all patients had given written informed consent to be included in the registry.

The analyses were conducted as part of the German innovation fund project ‘CED-KQN Big Data–eHealth: Improving the health care of children and adolescents with inflammatory bowel diseases’.

## Results

**Figure 1:**
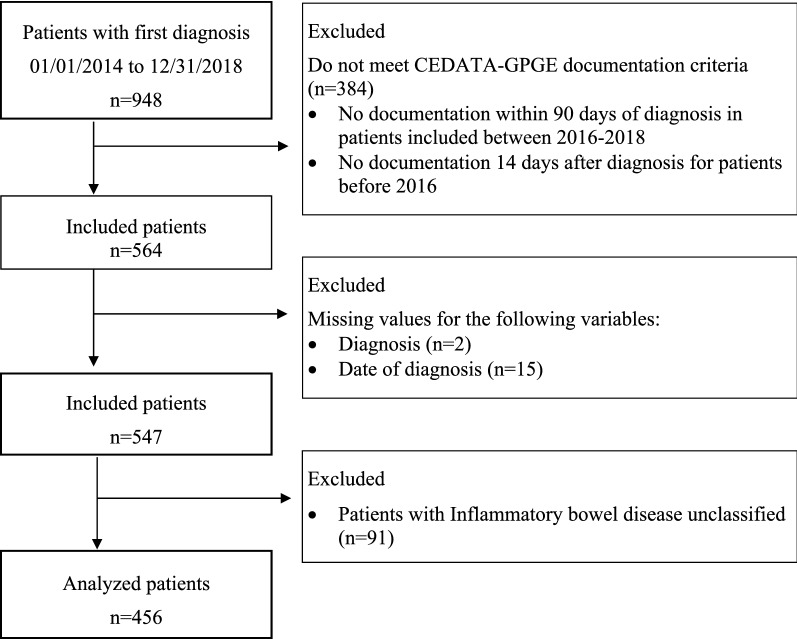
Flow chart of analyzed children and adolescents with IBD in the patient registry CEDATA-GPGE.

A total of n = 456 children from 33 pediatric gastroenterology centers in Germany (n = 28) and Austria (n = 5) were included in the analysis (Fig. 1). The minimum age of diagnosis was 1.7 years and the maximum age of diagnosis was 17.7 years.

### Description of patient characteristics

Table 1 shows the patient characteristics by diagnosis. CD was diagnosed in 258 children (56.6%). The median age of children with CD was 13.6 years (interquartile range (IQR): 11.2–15.2) and 41.9% of the children (n = 108) were female. UC was diagnosed in 198 children (43.4%). The median age of children with UC was 13.1 years (IQR 10.5–14.6), with 46.0% (n = 91) female children.

**Table 1:** Patient characteristics (sex, age, diagnostic delay) in total and by diagnosis.

	Total	Crohn’s disease	Ulcerative colitis	*p* value
Patients (n (%))	456 (100)	258 (100)	198 (100)	
Sex (n (%))				0.38
Boys	199 (43.6)	108 (41.9)	91 (46.0)	
Girls	257 (56.4)	150 (58.1)	107 (54.0)	
Age, in years (median (IQR))	13.3 (10.9–15.0)	13.6 (11.2–15.2)	13.1 (10.5–14.6)	0.16
Diagnostic delay, in months (median (IQR))	3.3 (1.8–6.5)	4.1 (2.1–7.0)	2.4 (1.2–5.1)	< 0.001

*IQR* interquartile range.

The three most common initial symptoms in CD were abdominal pain (76.4%, n = 197), diarrhea (67.8%, n = 175), and weight stagnation or loss (59.3%, n = 153). In children with UC, the three most common symptoms were visible blood in stool (83.3%, n = 165), diarrhea (79.8%, n = 158), and abdominal pain (72.2%, n = 143). A diagnosis of UC (median: 2.4 months; IQR: 1.2–5.1) is associated with a shorter diagnostic delay than CD (median: 4.1 months; IQR: 2.1–7.0; *P* < 0.001).

### Factors influencing diagnostic delay

Cox regression showed that children with UC had a significantly higher chance of early diagnosis, if the symptom abdominal pain at night occurred (HR = 1.80; 95% CI 1.05–3.10; *P* = 0.03), if video capsule endoscopy was performed (HR = 2.51; 95% CI 1.11–5.71; *P* = 0.03) or if the onset of first symptoms was late in the observation period in 2017 (HR = 1.85; 95% CI 1.15–2.97; *P* = 0.01) or 2018 (HR = 2.33; 95% CI 1.33–4.09; *P* = 0.003) (Table 2). The symptom abdominal pain (HR = 0.70; 95% CI 0.51; 0.96; *P* = 0.03) was associated with later diagnosis. Younger children tended to be diagnosed faster, but this effect was not significant (HR = 1.43; 95% CI 0.94–2.18; *P* = 0.10). The disease extent for UC did not have a significant effect on time to diagnosis.

**Table 2:** Univariate analysis of factors influencing diagnostic delay (in months) in children and adolescents with Ulcerative colitis (Hazard ratio < 1: longer, hazard ratio > 1: shorter).

	N	Median (25%–75%)	HR (95% CI)
**Demographics**			
Sex (*P* = 0.35)	198		
Boys	91	2.3 (1.1–5.8)	1.00
Girls	107	2.4 (1.3–5.1)	1.15 (0.86, 1.52)
Age at onset of symptoms (*P* = 0.12)	198		
0–9 years	38	2.0 (1.0–6.5)	1.43 (0.94, 2.18)
10–12 years	57	2.3 (1.2–3.9)	0.92 (0.61, 1.40)
13–14 years	57	3.0 (1.6–5.5)	1.03 (0.67, 1.59)
15–17 years	46	3.1 (1.0–5.3)	1.00
**Presenting symptoms**			
Abdominal pain (*P* = 0.03)	198		
No	55	2.1 (1.1–4.1)	
Yes	143	2.6 (1.3–6.0)	0.70 (0.51, 0.96)
Visible blood in stool (*P* = 0.77)	198		
No	33	3.1 (1.5–5.0)	
Yes	165	2.3 (1.2–5.1)	1.06 (0.73, 1.54)
Diarrhea (*P* = 0.72)	198		
No	40	3.0 (2.0–5.0)	
Yes	158	2.3 (1.1–5.2)	1.07 (0.75, 1.51)
Weight stagnation /weight loss (*P* = 0.64)	198		
No	119	2.4 (1.5–5.6)	1.00
Yes	79	2.2 (1.1–5.0)	1.07 (0.80, 1.43)
Stool (*P* = 0.16)	174		
Formed	47	3.1 (2.0–6.1)	1.00
Pulpy	60	3.3 (2.0–6.4)	1.01 (0.69, 1.48)
Liquid	67	1.5 (0.9–4.0)	1.36 (0.94, 1.99)
Abdominal pain at night (*P* = 0.03)	74		
No	56	2.4 (1.5–6.1)	1.00
Yes	18	1.6 (0.7–3.3)	1.80 (1.05, 3.10)
Abdominal pain (*P* = 0.54)	174		
None	57	2.1 (1.1–5.0)	1.00
Mild	49	3.2 (2.0–6.1)	0.76 (0.52, 1.12)
Moderate	57	2.3 (1.0–4.6)	0.93 (0.64, 1.35)
Severe	11	1.7 (0.7–3.9)	1.01 (0.52, 1.95)
**Disease phenotype**			
Abdominal findings (*P* = 1.00)	180		
Normal	121	2.4 (1.2–5.1)	1.00
Conspicuous	59	2.4 (1.4–5.4)	1.00 (0.73, 1.34)
Extraintestinal manifestations (*P* = 0.78)	183		
No	161	2.2 (1.2–5.0)	1.00
Yes	22	3.4 (1.4–6.7)	0.94 (0.60, 1.47)
Perianal disease (*P* = 0.89)	198		
No	192	2.4 (1.2–5.1)	1.00
Yes	6	3.2 (1.5–5.8)	0.94 (0.42, 2.13)
Disease extent (*P* = 0.62)	177		
Ulcerative proctitis	6	4.5 (3.2–12.7)	1.00
Left-sided UC	26	2.5 (1.0–5.1)	1.93 (0.79, 4,72)
Extensive	12	2.2 (1.5–4.2)	2.08 (0.78, 5.56)
Pancolitis	133	2.3 (1.2–5.0)	1.67 (0.73, 3.78)
**Diagnostic procedures**			
Oesophagoduodenoscopy (*P* = 0.90)	189		
No	63	2.7 (1.1–5.6)	1.00
Yes	126	2.3 (1.3–5.2)	1.02 (0.75, 1.38)
MR enterography (*P* = 0.30)	182		
No	138	2.3 (1.1–5.5)	1.00
Yes	51	2.6 (1.5–5.1)	1.19 (0.86, 1.65)
Ileocoloscopy (*P* = 0.36)	189		
No	109	2.7 (1.1–5.8)	1.00
Yes	80	2.3 (1.5–4.9)	1.15 (0.86, 1.54)
Histology lower gastrointestinal tract (*P* = 0.68)	173		
No	60	2.5 (1.0–5.5)	1.00
Yes	113	2.3 (1.3–5.2)	0.94 (0.68, 1.28)
Histology upper gastrointestinal tract (*P* = 0.66)	173		
No	72	2.4 (1.0–5.9)	1.00
Yes	101	2.3 (1.3–5.0)	1.07 (0.79, 1.45)
Colonoscopy (*P* = 0.74)	189		
No	118	2.5 (1.6–5.2)	
Yes	71	2.0 (1.0–5.8)	0.95 (0.70, 1.28)
Video capsule endoscopy (*P* = 0.03)	198		
No	183	2.4 (1.3–5.4)	1.00
Yes	6	0.9 (0.7–3.3)	2.51 (1.11, 5.71)
**Other factors**			
Center size* (*P* = 0.52)	170		
Small	80	2.5 (1.0–5.2)	1.00
Medium	35	2.3 (1.8–4.9)	0.94 (0.63, 1.40)
Large	55	2.3 (1.0–6.1)	0.82 (0.57, 1.16)
Time period from onset of symptoms (*P* < 0.001)	198		
2014	33	2.1 (1.5–5.8)	1.00
2015	47	3.5 (2.0–6.1)	0.83 (0.53, 1.30)
2016	58	3.2 (1.5–8.0)	0.79 (0.51, 1.23)
2017	39	1.6 (0.8–2.7)	1.85 (1.15, 2.97)
2018	21	1.6 (1.1–2.3)	2.33 (1.33, 4.09)

*small =  < 25 PIBD patients per year; medium = 25–100 PIBD patients per year; large =  > 100 PIBD patients per year.

In CD, children had a significantly higher chance of early diagnosis, if the onset of first symptoms was late in the observation period in 2018 (HR = 2.38; 95% CI 1.38–4.10; *P* = 0.002) (Table 3). There were no other parameters with a significant effect on diagnostic delay.

**Table 3:** Univariate analysis of factors influencing diagnostic delay (in months) in children and adolescents with Crohn’s disease (Hazard ratio < 1: longer, hazard ratio > 1: shorter).

	N	Median (25%–75%)	HR (95% CI)
**Demographics**			
Sex (*P* = 0.28)	258		
Boys	150	4.1 (2.0–6.8)	1.00
Girls	108	4.1 (2.3–8.5)	0.87 (0.68, 1.12)
Age at onset of symptoms (*P* = 0.33)	258		
0–9 years	32	4.0 (2.3–6.0)	1.15 (0.76, 1.75)
10–12 years	77	4.0 (2.0–6.6)	1.18 (0.85, 1.64)
13–14 years	77	4.5 (2.1–7.9)	0.89 (0.65, 1.23)
15–17 years	72	3.5 (2.2–7.1)	1.00
**Presenting symptoms**			
Abdominal pain (*P* = 0.26)	258		
No	61	4.4 (2.0–6.6)	
Yes	197	4.0 (2.1–7.1)	0.85 (0.63, 1.13)
Visible blood in stool (*P* = 0.29)	258		
No	159	4.2 (2.1–7.0)	
Yes	99	3.5 (2.0–7.1)	1.15 (0.89, 1.48)
Diarrhea (*P* = 0.48)	258		
No	83	4.5 (2.2–7.1)	
Yes	175	4.0 (2.0–7.0)	1.10 (0.85, 1.43)
Weight stagnation /weight loss (*P* = 0.73)	258		
No	105	3.6 (2.0–7.0)	1.00
Yes	153	4.3 (2.1–7.0)	1.05 (0.81, 1.34)
Stool (*P* = 0.86)	245		
Formed	99	4.1 (2.0–6.8)	1.00
Pulpy	71	3.7 (2.0–6.1)	1.03 (0.76, 1.41)
Liquid	75	4.0 (2.0–7.1)	0.95 (0.70, 1.28)
Fistula (*P* = 0.37)	258		
No	231	4.1 (2.1–6.9)	1.00
Yes	27	6.1 (1.3–8.9)	0.83 (0.56, 1.24)
Abdominal pain at night (*P* = 0.53)	106		
No	85	3.5 (2.2–7.1)	1.00
Yes	21	4.0 (1.3–6.6)	0.85 (0.52, 1.40)
Abdominal pain (*P* = 0.27)	233		
None	77	3.7 (2.0–6.1)	1.00
Mild	53	4.7 (2.2–7.0)	0.76 (0.54, 1.09)
Moderate	90	4.0 (2.1–6.8)	0.83 (0.61, 1.13)
Severe	13	6.8 (1.2–11.4)	0.62 (0.34, 1.11)
**Disease phenotype**			
Abdominal findings (*P* = 0.89)	240		
Normal	144	4.0 (2.0–7.1)	1.00
Conspicuous	96	4.1 (2.2–6.7)	0.98 (0.76, 1.27)
Extraintestinal manifestations (*P* = 0.18)	241		
No	201	4.0 (2.0–6.8)	1.00
Yes	40	4.8 (2.3–9.9)	0.79 (0.56, 1.12)
Perianal disease (*P* = 0.97)	258		
No	219	4.0 (2.0–6.9)	1.00
Yes	39	5.0 (2.1–7.6)	0.99 (0.70, 1.40)
Ileal Crohn (*P* = 0.22)	225		
No	209	4.2 (2.2–7.1)	1.00
Yes	16	3.5 (1.6–7.8)	1.11 (0.67, 1.84)
**Diagnostic procedures**			
Oesophagoduodenoscopy (*P* = 0.66)	253		
No	76	3.9 (2.0–6.5)	1.00
Yes	177	4.1 (2.2–7.0)	0.94 (0.72, 1.24)
MR enterography (*P* = 0.62)	253		
No	134	3.6 (2.0–7.1)	1.00
Yes	119	4.4 (2.3–6.8)	0.94 (0.73, 1.20)
Ileocoloscopy (*P* = 0.48)	253		
No	143	3.5 (2.0–6.9)	1.00
Yes	110	4.8 (2.2–7.0)	0.91 (0.71, 1.17)
Histology lower gastrointestinal tract (*P* = 0.78)	213		
No	69	4.1 (2.0–7.6)	1.00
Yes	144	4.1 (2.2–6.9)	1.04 (0.78, 1.39)
Histology upper gastrointestinal tract (*P* = 0.82)	213		
No	66	4.0 (2.0–7.6)	1.00
Yes	147	4.2 (2.3–6.9)	1.04 (0.77, 1.39)
Colonoscopy (*P* = 0.62)	253		
No	173	4.1 (2.0–6.8)	1.00
Yes	80	3.8 (2.2–7.6)	0.93 (0.72, 1.22)
Video capsule endoscopy (*P* = 0.22)	253		
No	245	4.1 (2.0–6.8)	1.00
Yes	8	6.0 (3.1–12.5)	0.64 (0.32, 1.30)
**Other factors**			
Center size* (*P* = 0.29)	211		
Small	59	4.1 (2.3–7.1)	1.00
Medium	68	3.8 (2.3–6.8)	1.29 (0.90, 1.85)
Large	84	3.4 (1.6–6.9)	1.27 (0.91, 1.78)
Time period from onset of symptoms (*P* = 0.002)	258		
2014	48	4.2 (2.1–8.6)	1.00
2015	62	5.1 (2.7–9.1)	0.87 (0.60, 1.27)
2016	63	3.7 (2.0–7.1)	1.39 (0.95, 2.04)
2017	66	4.1 (2.2–6.8)	1.35 (0.93, 1.98)
2018	19	2.8 (1.8–3.5)	2.38 (1.38, 4.10)

*small =  < 25 PIBD patients per year; medium = 25–100 PIBD patients per year; large =  > 100 PIBD patients per year.

## Discussion

There are significant differences in diagnostic delay between the diagnoses of UC and CD. This finding is in line with other studies, that found CD associated with longer diagnostic delay as well^1,9,16–18^. Within CD, ileal disease was not associated with delayed diagnosis, which is in contrast to Timmer et al.^16^. However, in our analysis, ileal disease occurred in only 16 of 258 children and adolescents with CD, of whom four had blood in stool.

Blood in stool as a distinctive symptom of UC may lead to a diagnosis more quickly than, for example, growth retardation typical of CD, which has a much broader differential diagnosis in general pediatrics.

Abdominal pain is among the most common chronic pain symptoms in children and adolescents in Germany^19^. The sole presence of common symptoms such as abdominal pain leads to a delay in diagnosis^1^. Abdominal pain at night is considered a “red flag” in the algorithm for pediatric functional abdominal pain and thus leads to a faster investigation of organic causes.

Video capsule endoscopy is not routinely used in UC, but only in a very small proportion of patients. However, it can help to clarify initial colitis not typical for UC and differentiate towards L2 CD. The faster diagnosis of UC when video capsule endoscopy is performed is probably related to its mainly exclusive use by larger centers. The relation between diagnostic delay and the center’s structural characteristics was also found as a center effect by Timmer et al.^16^. Turner et al. found that a center effect is caused by the varying availability of facilities, personnel, management, supportive services, etc. and that there is a trend for increased availability with increased patient volume at the centers^20^. However, univariate analysis did not show any significant difference between smaller and larger, more specialized centers. This could be due to, for example, a larger number of complex cases at larger centers.

While many studies report diagnostic delay approaching one year in CD^10,21,22^, in previous analyses, diagnostic delay in CEDATA-GPGE patients was found to be shorter, with 50% of children receiving their diagnosis within four months^16^. Other registry data analyses revealed comparable results with 2–4 months in the French EPIMAD study, 3 months in Spain (with a significant share of patients over 1 year), 4–5 months in Norway and the UK, and 6–10 months in the Italian registry^22–26^.

Even though 2017 and 2018 were associated with earlier diagnosis compared to 2014, there is no relevant improvement of median diagnostic delay over the last ten years of the registry. In the period 2004–2009, there was a diagnostic delay of median 4 months in patients with PIBD^1,16^. In the period 2004–2014, there was a diagnostic delay of 6 months in CD and 4 months in UC^1^. However, patients with delays more than six months seem to be reduced compared to CEDATA-GPGE data from 2011, reflecting recent advances in pediatric IBD care^16^. This trend is also reported from other analyses, e.g. Finland^11^.

In Germany and Austria, children and adolescents receive regular preventive medical care from family medicine or pediatricians, all relevant procedures are covered by ubiquitous health insurance. There is no relevant barrier to diagnosing IBD and most delay results from later referral and pre-specialist consultation. In some areas of Germany, coverage of pediatric gastroenterology specialist care still requires families to travel long distances, thus hindering referral in some cases.

For five of 35 selected parameters a significant but small effect on diagnostic delay could be shown. However, the number of children and adolescents and participating gastroenterological centers varies (e.g. 2017:21 centers vs. 2018:15 centers) and the numbers within the respective parameters are partly very small (e.g. video capsule endoscopy in UC: n = 6).

Another limitation of the analysis is that only the time interval between the date of the first visit to the specialized center and the date of diagnosis can be considered in detail, because the information about the time period before the first visit to the center with e.g. contacts with outpatient pediatricians, can only be assessed by asking the children and adolescents or their parents. Other limitations include varying diagnostic approaches in the participating centers and data acquisition from specialized centers. Only pediatric gastroenterologists document in the registry. Non-pediatric gastroenterologists (e.g. internist gastroenterologists) are not actively recruited. CEDATA-GPGE is not population-based, therefore some referral bias is likely.

The strength of the study is the comparatively high number of patients, the clinical data provided prospectively by physicians in charge and not by retrospective chart review, and the follow-up data in the registry, which can be used for further in-depth analyses.

## Conclusion

In conclusion, the time between initial presentation and a confirmed diagnosis varies for Crohn’s disease and Ulcerative colitis considerably. The threshold for investigating pediatric-onset IBD non-invasively also with atypical findings and referral to specialized centers needs to be lowered to reduce diagnostic delay.

## Supplementary Information

Supplementary Information.

## Data Availability

The data that support the findings of this study are available from the corresponding author upon reasonable request.

## References

[1] Buderus S (2015). Inflammatory bowel disease in pediatric patients: Characteristics of newly diagnosed patients from the CEDATA-GPGE Registry. Dtsch Arztebl Int..

[2] Benchimol EI (2011). Epidemiology of pediatric inflammatory bowel disease: A systematic review of international trends. Inflamm. Bowel Dis..

[3] Sýkora J (2018). Current global trends in the incidence of pediatric-onset inflammatory bowel disease. World J. Gastroenterol..

[4] Kuenzig E (2022). Twenty-first century trends in the global epidemiology of pediatric-onset inflammatory bowel disease: Systematic review. Gastroenterology.

[5] Wittig R, Albers L, Koletzko S, Saam J, Kries RV (2018). Pediatric chronic inflammatory bowel disease in a German statutory health INSURANCE—Incidence rates from 2009–2012. J. Pediatr. Gastroenterol. Nutr..

[6] Ward L (2017). Musculoskeletal health in newly diagnosed children with Crohn’s disease. Osteoporos. Int..

[7] Assa A, Ish-Tov A, Rinawi F, Shamir R (2015). School attendance in children with functional abdominal pain and inflammatory bowel diseases. J. Pediatr. Gastroenterol. Nutr..

[8] Wong K, Isaac DM, Wine E (2021). Growth delay in inflammatory bowel diseases: Significance, causes, and management. Dig. Dis. Sci..

[9] Sulkanen E, Repo M, Huhtala H, Hiltunen P, Kurppa K (2021). Impact of diagnostic delay to the clinical presentation and associated factors in pediatric inflammatory bowel disease: A retrospective study. BMC Gastroenterol..

[10] Sawczenko A, Ballinger AB, Savage MO, Sanderson IR (2006). Clinical features affecting final adult height in patients with pediatric-onset Crohn’s disease. Pediatrics.

[11] Gower-Rousseau C (2009). The natural history of pediatric ulcerative colitis: A population-based cohort study. Am. J. Gastroenterol..

[12] Levine A (2014). ESPGHAN revised porto criteria for the diagnosis of inflammatory bowel disease in children and adolescents. J. Pediatr. Gastroenterol. Nutr..

[13] Levine A (2011). Pediatric modification of the montreal classification for inflammatory bowel disease: The Paris classification. Inflamm. Bowel Dis..

[14] Hoffmann W (2019). Guidelines and recommendations for ensuring Good Epidemiological Practice (GEP): A guideline developed by the German Society for Epidemiology. Eur. J. Epidemiol..

[15] World Medical Association (2013). WMA Declaration of Helsinki—Ethical principles for medical research involving human subjects. JAMA.

[16] Timmer A (2011). Childhood onset inflammatory bowel disease: predictors of delayed diagnosis from the CEDATA German-language pediatric inflammatory bowel disease registry. J. Pediatr..

[17] Kern I (2021). Incidence trends of pediatric onset inflammatory bowel disease in the years 2000–2009 in Saxony, Germany–first results of the Saxon Pediatric IBD Registry. PLoS ONE.

[18] Cantoro L (2017). The time course of diagnostic delay in inflammatory bowel disease over the last sixty years: An Italian multicentre study. J. Crohns Colitis.

[19] Schwille IJD, Giel KE, Ellert U, Zipfel S, Enck P (2009). A community-based survey of abdominal pain prevalence, characteristics, and health care use among children. Clin. Gastroenterol. Hepatol..

[20] Turner D (2017). Quality items required for running a paediatric inflammatory bowel disease centre: An ECCO paper. J. Crohns Colitis.

[21] Grieci T, Butter A (2009). The incidence of inflammatory bowel disease in the pediatric population of southwestern Ontario. J. Pediatr. Surg..

[22] Bentsen B, Moum B, Ekbom A (2002). Incidence of inflammatory bowel disease in children in southeastern Norway: A prospective population-based study 1990–94. Scand. J. Gastroenterol..

[23] Newby EA (2008). Natural history of paediatric inflammatory bowel diseases over a 5-year follow-up: A retrospective review of data from the register of paediatric inflammatory bowel diseases. J. Pediatr. Gastroenterol. Nutr..

[24] Arcos-Machancoses J (2015). Description and study of risk factors for the diagnostic delay of pediatric inflammatory bowel disease. Anales de Pediatría (English Edition).

[25] Castro M (2008). Inflammatory bowel disease in children and adolescents in Italy: Data from the Pediatric National IBD Register (1996–2003). Inflamm. Bowel Dis..

[26] Auvin S (2005). Incidence, clinical presentation and location at diagnosis of pediatric inflammatory bowel disease: A prospective population-based study in Northern France (1988–1999). J. Pediatr. Gastroenterol. Nutr..

