# Supplementary material for: Diagnostic delay in children with inflammatory bowel disease in the German-Austrian patient registry CEDATA-GPGE 2014–2018
Source: Sci Rep. 2022 Dec 7;12:21162. doi: 10.1038/s41598-022-25487-6 (PMC9729560; doi:10.1038/s41598-022-25487-6)
Supplement: Supplementary file 1 — Supplementary Information. [file 41598_2022_25487_MOESM1_ESM.pdf]

CEDATA

CHRONIC INFLAMMATORY BOWEL DISEASE (IBD) OF CHILDS AND ADOLESCENTS

INITIAL REPORTING FORM

[| |] - [| | | |]
Patient's identification number

Patient

Sex [ ] | Date of birth (MM/YYYY) [| | | | |] | Date of investigation (DD/MM/YYYY) [| | | | | | |]

Country of birth [ ]

Postal code [| |]

Basis data

Type of Presentation	Reason visit	Diagnosis	other chronic diseases ○ no ○ yes		
○ outpatient	○ planned	○ Crohn's disease	☐ allergy	☐ autoimmune disease	height [ ]
○ day ward	○ acute	○ Ulcerative colitis	☐ proven immunodeficiency	☐ further malignant disease	weight [ ]
○ inpatient		○ unspecific CED	☐ other serious disease		

Diagnosis & symptoms

Diagnosis by:	Diagnosis in:	Symptoms until diagnosis ○ no ○ yes			
○ pediatric gastroenterologist	○ own clinic/hospital	☐ fever	☐ reduced performance	☐ lip-/mouthinvolvement	☐ nausea/vomiting
○ gastroenterologist	○ other hospital	☐ anemia	☐ other fistula	☐ visible blood in stool	☐ abdominal pain
○ pediatric surgeon/surgeon	○ other clinic	☐ diarrhea	☐ loss of appetite	☐ weightstagnancy/-loss	☐ perianal lesion
		☐ disturbance of growth	☐ other: [ ]		

date of diagnosis (DD/MM/YYYY) [| | | | | | |] date of first symptoms (DD/MM/YYYY) [| | | | | | |]

Family history

mother's height [ ] measured in ambulance: ○ no ○ yes

mother's country of birth [ ]

mother's date of birth (MM/YYYY) [| | | | |]

father's height [ ] measured in ambulance: ○ no ○ yes

father's country pf birth [ ]

Vaccination suitable STIKO:	Number of siblings:		IBD in family: ○ no ○ yes	
○ complete	(half)sisters	[ ]	IBD mother	○ CD ○ UC ○ IBD-U ○ no IBD
○ not complete	(half)brothers	[ ]	IBD father	○ CD ○ UC ○ IBD-U ○ no IBD
○ not applicable			IBD siblings	○ CD ○ UC ○ IBD-U ○ no IBD
			IBD relatives	○ CD ○ UC ○ IBD-U ○ no IBD

Anamnesis until diagnosis

Condition	Appetite	Restrictions daily life	Fever	Defecation: Consistence	Defecation: Blood in stool	Defecation: Count	Abdominal pain
○ very good	○ good	○ no	○ no	○ shaped	○ no	by day [ ]	○ none
○ good	○ moderate	○ mild	○ yes	○ mushy	○ occasionally, little	by night [ ]	○ lightly
○ medium	○ bad	○ obvious		○ liquid	○ mostly, little		○ moderately
○ bad					○ much		○ severe
○ very bad							☐ by night

Examination

Abdominal finding	Anal finding	Perianal eczema	Oral aphtae ulcers	cheilitis	extraintestinal Symptoms ○ no ○ yes	
○ inconspicuous	☐ inconspicuous/irritation-free skin-tags	○ no	○ no	○ no	☐ eye	☐ skin
○ conspicuous	☐ rhagade/fissure	○ yes	○ yes	○ yes	☐ liver/biliary tract/pancreas	☐ PSC/overlap
☐ pressure pain	☐ inactive fistula				☐ acute pancreatitis	☐ nephritis
☐ resistance	☐ secreting fistula/abcess inflammatory induration				☐ joint: inflammation peripheral	☐ spinal column
☐ defense	☐ multiple/inflammatory skin-tags				☐ joint: pain peripheral	☐ others

Tanner's stages PH(1-5) [ ] B(1-5) [ ] testicular volume (ml) [|] menarche: ○ no ○ yes [| | |] (year)

laboratory

hemoglobin	___	○mmol/l ○g/dl	CRP	___	○mg/dl ○mg/l	albumin	___	○g/dl ○g/l
hematocrit	___	○% ○l/l	ESR	___	mm/h	creatinine	___	○mg/dl ○µmol/l
MCV	___	○µm³ ○fl	ALAT (GPT)	___	○U/l ○µmol/(l*s)	calprotectin	___	○mg/l ○mg/kg
thrombocytes	___	○1/µl ○Gpt/l	Gamma-GT	___	○U/l ○µmol/(l*s)			
leucocytes	___	○1/µl ○Gpt/l	lipase	___	○U/l ○µmol/(l*s)			

rare laboratory: ○ no ○ yes **(see additional sheet)**

CHRONIC INFLAMMATORY BOWEL DISEASE (IBD) OF CHILDS AND ADOLESCENTS

INITIAL REPORTING FORM

[| |] - [| | | |]
Patient's identification number

apparative diagnostics

apparative diagnostics ○ not performed ○ performed

☐ esophagogastroduodenoscopy ☐ MRT-enterography ☐ ileocoloscopy ☐ colonoscopy ☐ rectosigmoidoscopy
☐ histology lower gastrointestinal tract ☐ X-ray small intestine ☐ ultrasound ☐ bone densitometry ☐ others:
☐ histology upper gastrointestinal tract ☐ CT abdomen ☐ video capsule endoscopy ☐ liver biopsy

localization

localization ☐ mouth ☐ esophagus ☐ stomach ☐ duodenum ☐ remaining small intestine ☐ term. ileum
○ inconspicuous ☐ caecum ☐ colon asc. ☐ colon trans. ☐ colon desc. ☐ sigmoid colon ☐ rectum ☐ anus
overall assessment:

Comp.

complications ○ no ○ yes ○ unknown

☐ perianal abscess ☐ stenosis ☐ perianal fistula ☐ other type of fistula ☐ other type of abscess

Surgery

Surgery ○ no ○ yes ○ planned surgery date of surgery:

Indication	Type of surgery	Type of fistula * (referring to surgery fistula)	Localization ° (referring to surgery stenosis)
☐ conglomerate tumor<br>☐ abscess (except perianal)<br>☐ stenosis ° ☐ megacolon<br>☐ perforation ☐ fistula (expect perianal) *<br>☐ suture dehiscence<br>☐ bleeding<br>☐ perianal complications (fistula, abscess)<br>☐ others:	☐ ileocecal resection ☐ colectomy<br>☐ hemicolectomy right ☐ hemicolectomy left<br>☐ partial colon resection ☐ rectal resection<br>☐ gastrojejunostomy ☐ partial small intestine resection<br>☐ fistula drainage ☐ exploration<br>☐ stricturoplasty ☐ fistula/abscess cleavage<br>☐ balloon dilatation ☐ PEG-insertion with jejunal extension<br>☐ PEG-insertion ☐ polypectomy<br>☐ stoma insertion<br>☐ others:	☐ rectovaginal<br>☐ enterovesical<br>☐ enterocutaneous<br>☐ enteroenteral<br>☐ enterocolistic<br>☐ ending blindly	☐ esophagus<br>☐ stomach<br>☐ duodenum<br>☐ prox. ileum, Jejunum<br>☐ term. ileum<br>☐ colon<br>☐ rectum

Induction therapy

Induction therapy ○ no ○ yes

1st month	2nd month weight: height:	3rd month weight: height:
☐ antibiotics * ☐corticoids(not budesonide)	☐ antibiotics * ☐corticoids(not budesonide)	☐ antibiotics * ☐corticoids(not budesonide)
☐ tacrolimus ☐ 6-mercaptopurine	☐ tacrolimus ☐ 6-mercaptopurine	☐ tacrolimus ☐ 6-mercaptopurine
☐ budesonide ☐ methotrexate	☐ budesonide ☐ methotrexate	☐ budesonide ☐ methotrexate
☐ vitamin D ☐ adalimumab	☐ vitamin D ☐ adalimumab	☐ vitamin D ☐ adalimumab
☐ azathioprine ☐ other biologicals<br>○ allopurinol name:	☐ azathioprine ☐ other biologicals<br>○ allopurinol name:	☐ azathioprine ☐ other biologicals<br>○ allopurinol name:
☐ iron ○ ☐ Cyclosporin A<br>p.o ○i.v. ○n.s. ○p.o ○i.v. ○n.s.	☐ iron ○ ☐ Cyclosporin A<br>p.o ○i.v. ○n.s. ○p.o ○i.v. ○n.s.	☐ iron ○ ☐ Cyclosporin A<br>p.o ○i.v. ○n.s. ○p.o ○i.v. ○n.s.
☐ calcium ☐ steroid pulse therapy	☐ calcium ☐ steroid pulse therapy	☐ calcium ☐ steroid pulse therapy
☐nutritional therapy ☐ complentary medicine<br>○exclusively ○partially ☐incense ☐fish-oil ☐lecithin<br>○supplementary ☐probiotics	☐nutritional therapy ☐ complentary medicine<br>○exclusively ○partially ☐incense ☐fish-oil ☐lecithin<br>○supplementary ☐probiotics	☐nutritional therapy ☐ complentary medicine<br>○exclusively ○partially ☐incense ☐fish-oil ☐lecithin<br>○supplementary ☐probiotics
☐ sulfasalazine ☐ vedolizumab [1]<br>☐ mesalazine ☐ ustekinumab [1]	☐ sulfasalazine ☐ vedolizumab [1]<br>☐ mesalazine ☐ ustekinumab [1]	☐ sulfasalazine ☐ vedolizumab [1]<br>☐ mesalazine ☐ ustekinumab [1]
☐ golimumab [1] ☐ special diet<br>☐ tofacitinib [1]	☐ golimumab [1] ☐ special diet<br>☐ tofacitinib [1]	☐ golimumab [1] ☐ special diet<br>☐ tofacitinib [1]
☐ rectal therapy ○5-ASA mesalazine<br>○ corticoids ○ budesonide	☐ rectal therapy ○5-ASA mesalazine<br>○ corticoids ○ budesonide	☐ rectal therapy ○5-ASA mesalazine<br>○ corticoids ○ budesonide

* see additional sheet **1 cave:** off-label use

side effects

Side effects ○ no ○ yes

☐ acne ☐ depressiveness ☐ alopecia ☐ increased levels of lipases ☐ increased levels of transaminases
☐ abdominal pain ☐ diarrhea ☐ hypertrichosis ☐ acute allergic reaction ☐ hypertension
☐ vomiting/nausea ☐ headaches ☐ pancreatitis ☐ personality changes ☐ Cushing's disease
☐ glaucoma ☐ leukopenia ☐ thrombocytopenia ☐ skin changes ☐ nephritis/increased levels of creatinine

conclusion

Medical overall assessment

○ remission ○ mild activity ○ moderate activity ○ severe activity

CEDATA

CHRONIC INFLAMMATORY BOWEL DISEASE (IBD) OF CHILDS AND ADOLESCENTS

DOCUMENTATIONFORM

Patient's identification number: [| |] - [| | | |]

Basis data

Date of presentation (DD/MM/YYYY)

Date of last presentation(DD/MM/YYYY)

Number of undocumented presentations

Type of presentation	Reason	Diagnosis	Other chronic diseases ○ no ○ yes		
○ outpatient	○ planned	○ Crohn's disease	☐ allergy	☐ autoimmune disease	height
○ day ward	○ acute	○ Ulcerative colitis	☐ proven immunodeficiency	☐ further malignant disease	weight
○ inpatient		○ unspecific IBD	☐ other serious disease		

Anamnesis

Condition	Appetite	Restrictions daily life	Fever	Defecation: consistence	Defecation: blood in stool	Defecation: Count	Abdominal pain	vaccination since last inves-
○ very good	○ good	○ no	○ no				○ none	○ yes
○ good	○ moderate	○ mild	○ yes	○ shaped	○ no	by day	○ lightly	○ no
○ medium	○ bad	○ obvious		○ mushy	○ occationally, little	by night	○ moderately	Which?
○ bad		school absence days		○ liquid	○ mostly, little		○ severe	
○ very bad					○ much		☐ by night	

Examination

Abdominal finding ○ inconspicuous ○ conspicuous	Anal finding	Perianal eczema	Oral aphtae ulcers	Cheilitis	extraintestinal symtoms ○ no ○ yes	
	☐ inconspicuous/irritation-free skin-tags	○ no	○ no	○ no	☐ eye	☐ skin
☐ pressure pain	☐ rhagade/fissure	○ yes	○ yes	○ yes	☐ liver/biliary tract/pancreas	☐ PSC/overlap
☐ resistance	☐ inactive fistula				☐ acute pancreatitis	☐ nephritis
☐ defense	☐ secreting fistula/abscess inflam-matory induration				☐ joint: inflammation peripheral	☐ spinal column
☐ stoma	☐ multiple/inflammatory skin-tags				☐ joint: pain peripheral	☐ others

Tanner' stages PH(1-5) [ ] B(1-5) [ ] testicular volume (ml) [ ] menarche: ○ no ○ yes [ ] (year)

laboratory

hemoglobin	○mmol/l ○g/dl	CRP	○mg/dl ○mg/l	albumin	○g/dl ○g/l	seldom laboratory:
hematocrit	○% ○l/l	ESR	mm/h	creatinine	○mg/dl ○µmol/l	○ no
MCV	○µm³ ○fl	ALAT (GPT)	○U/l ○µmol/(l*s)	calprotectin	○mg/l ○mg/kg	○ yes
thrombocytes	○1/µl ○Gpt/l	gamma-GT	○U/l ○µmol/(l*s)			(see additi-onal sheet)
leucocytes	○1/µl ○Gpt/l	lipase	○U/l ○µmol/(l*s)			

apparative diagnostics ○ not performed ○ performed

☐ esophagogastroduodenoscopy	☐ MRT-enterography	☐ ileokoloscopy	☐ colonoscopy	☐ rectosigmoidoscopy
☐ histology lower gastrointestinal tract	☐ X-ray small intestine	☐ ultrasound	☐ bone densitometry	☐ liver biopsy
☐ histology upper gastrointestinal tract	☐ CT abdomen	☐ video capsule endoscopy	☐ ERCP	☐ MRCP

☐ others:

Localization ○ inconspicuous

☐ mouth	☐ esophagus	☐ stomach	☐ duodenum	☐ remaining small intesine	☐ term. ileum	☐ pouch
☐ caecum	☐ colon asc.	☐ colon trans.	☐ colon desc.	☐ sigmoid colon	☐ rectum	☐ anus

☐ overall assessment:

Complications ○ no ○ yes

☐ CMV infection	☐ vomiting	☐ osteoporosis	☐ perianal fistula	☐ other type of fistula	☐ perianal abscess
☐ other type of abscess	☐ biliary calculi	☐ stenosis	☐ unreliable medication intake		

CEDATA

CHRONIC INFLAMMATORY BOWEL DISEASE (IBD) OF CHILDS AND ADOLESCENTS

DOCUMENTATIONFORM

[|] - [| | | |]

Patient's identification number

Surgery

Surgery ○ no ○ yes ○ planned surgery date of surgery:

**Indication**		**Type of surgery**		
☐ conglomerate tumor	☐ perforation	☐ ileocecal resection	☐ colectomy	☐ polypectomy
☐ stenosis (**see additional sheet**)	☐ megacolon	☐ hemicolectomy right	☐ pouch revision	☐ PEG-insertion
☐ fistula (not perianal) (**see additional sheet**)	☐ suture dehiscence	☐ hemicolectomy left	☐ rectal resection	☐ stoma insertion
☐ perianal complications (fistula, abscess)	☐ bleeding	☐ partial colon resektion	☐ adhesiolysis	☐ stoma revision
☐ refractory inflammation (therapy-resistant, chronic course)		☐ partial small intestine resection	☐ fistula drainage	☐ exploration
☐ colon carcinoma	☐ abscess (except perianal)	☐ gastrojejunostomy	☐ balloon dilatation	☐ stricturoplasty
☐ dysplasia/other malignancies: which?		☐ fistula/abscess cleavage	☐ PEG-insertion with jejunal extension	
☐ others:		others:		

Therapy

Therapy ○ no ○ yes

☐ sulfasalazine	start:	end:	dosis: mg/day	☐ trace elements:	☐ folic acid	☐ vitamin D
☐ mesalazine	start:	end:	dosis: mg/day		☐ vitamin B_{12}	☐ calcium
☐ azathioprine<br>○ Allopurinol	start:	end:	dosis: mg/day		☐ zinc	☐ selenium
☐ 6-mercaptopurine	start:	end:	dosis: mg/day	☐ comple-mentary medicine	☐ incense	☐ fish oil
☐ mycophenolate mofetil	start:	end:	dosis: mg/day		☐ lecithin	
☐ acid blockers	start:	end:	dosis: mg/day	☐ nuritional therapy	○ exclusively<br>○ supplementary	○ partially
☐ ursodeoxycholic acid	start:	end:	dosis: mg/day			
☐ loperamide	start:	end:	dosis: mg/day	☐ probiotics:	○ no	○ yes
☐ steroid pulse therapy	start:	end:	dosis: mg/day	☐ antibiotics:	○ no	○ yes (**s. add. sheet**)
☐ methotrexate	start:	end:	dosis: mg/week ○ s.c ○ p.o	○ no folic acid<br>○ 1mg folic acid for 5 days	○ 5mg folic acid single dosis	
☐ infliximab	start:	end:	dosis: mg	○ every _____week(s)		
☐ adalimumab	start:	end:	dosis: mg	○ every _____week(s)		
☐ other biologicals	start:	end:	dosis: mg	○ every _____week(s) name:		
☐ vedolizumab [1]	start:	end:	dosis: mg	○ every _____week(s)	[1] cave: off-label use	
☐ ustekinumab [1]	start:	end:	dosis: mg	○ every _____week(s)		
☐ golimumab [1]	start:	end:	dosis: mg	○ every _____week(s)		
☐ tofacitinib [1]	start:	end:	dosis: mg	○ every _____week(s)		
☐ cyclosporine A	start:	end:	dosis: mg/day	○ p.o	○ i.v.	○ n.s.
☐ iron	start:	end:	dosis: mg/dosis	○ p.o	○ i.v.	○ n.s.
☐ special diet	start:	end:	name:			
☐ rectal therapy	☐ 5-ASA mesalazine	☐ corticoids	☐ budesonide	☐ tacrolimus		

sys. coticoide ○no ○yes

☐ budesonide	start:	end:	dosis: mg/day	
☐ (methyl-)prednisolone	start:	end:	dosis: mg/day	maximum dose:
☐ hydrocortisone	start:	end:	dosis: mg/day	

Side effects

Side effects ○ no ○ yes

☐ depressiveness	☐ acne	☐ Cushing's disease	☐ increased levels of lipases	☐ increased levels of transaminases		☐ glaucoma
☐ abdominal pain	☐ diarrhea	☐ hypertrichosis	☐ acute allergic reaction	☐ hypertension	☐ alopecia	☐ leukopenia
☐ vomiting/nausea	☐ headaches	☐ thrombocytopenia	☐ personality changes	☐ nephritis/increased levels of creatinine		☐ pancreatitis

conclusion

**Medical overall assessment**	**Psychosocial therapy**	**Ophthalmologist appointment**
○ remission ○ mild activity ○ moderate activity ○ severe activity	○ no ○ yes	○ no ○ yes

CEDATA

CHRONIC INFLAMMATORY BOWEL DISEASE (IBD) OF CHILDS AND ADOLESCENTS

ADDITIONAL SHEET

Date of investigation (DD/MM/YYYY): ___ ___ ___

Patient's identification number: ___ - ___

Seldom laboratory

Immunodeficiency	TPMT Aktivity	6TG	MMP	Anti-HBS
O no O yes	O no O yes	O no O yes	O no O yes	O no O yes
	___________ Onmol/gHg*h	___________ Opmol/0,2 ml	___________ Opmol/8x10^8Eryth	U/ml
	OmU/L Onmol/ml x RBC*h	O pmol/8*10^8RBC	Onmol/gHg*h Opmol/0,2 ml	

Infliximab-level	Infliximab-antibody-level	Adalimumab-level	Adalimumab-antibody-level	Hepatitis B serology
O no O yes	O no O yes	O no O yes	O no O yes	O positive
µg/ml	AU/ml	µg/ml	AU/ml	O negative
date of sampeling:	date of sampeling:	date of sampeling:	date of sampeling:	O unknown

Hepatitis C serology	HBV-DNA-PCR	EBV	Tuberculosis skin test	Tuberculosis blood test
O positive	O positive	O positive	O positive	O positive
O negative	O negative	O negative	O negative	O negative

Antibiotic therapy

Antibiotic therapy O yes O no							
☐ amoxicillin	start:		end:		reason:		O p.o O i.v. O n.s.
☐ ampicillin	start:		end:		reason:		O p.o O i.v. O n.s.
☐ cefotaxime	start:		end:		reason:		O p.o O i.v. O n.s.
☐ cefpodoxime	start:		end:		reason:		O p.o O i.v. O n.s.
☐ ceftazolin	start:		end:		reason:		O p.o O i.v. O n.s.
☐ ceftriaxone	start:		end:		reason:		O p.o O i.v. O n.s.
☐ cefuroxime	start:		end:		reason:		O p.o O i.v. O n.s.
☐ ciprofloxacin	start:		end:		reason:		O p.o O i.v. O n.s.
☐ clarithromycin	start:		end:		reason:		O p.o O i.v. O n.s.
☐ clindamycin	start:		end:		reason:		O p.o O i.v. O n.s.
☐ cotrimoxazole	start:		end:		reason:		O p.o O i.v. O n.s.
☐ daptomycin	start:		end:		reason:		O p.o O i.v. O n.s.
☐ doxycycline	start:		end:		reason:		O p.o O i.v. O n.s.
☐ erythromycin	start:		end:		reason:		O p.o O i.v. O n.s.
☐ imipenem	start:		end:		reason:		O p.o O i.v. O n.s.
☐ linezolid	start:		end:		reason:		O p.o O i.v. O n.s.
☐ meropenem	start:		end:		reason:		O p.o O i.v. O n.s.
☐ metronidazole	start:		end:		reason:		O p.o O i.v. O n.s.
☐ paromomycin	start:		end:		reason:		O p.o O i.v. O n.s.
☐ piperacillin/sulbactan	start:		end:		reason:		O p.o O i.v. O n.s.
☐ piperacillin/tazobactam	start:		end:		reason:		O p.o O i.v. O n.s.
☐ penicillin	start:		end:		reason:		O p.o O i.v. O n.s.
☐ rifaximin	start:		end:		reason:		O p.o O i.v. O n.s.
☐ vancomycin	start:		end:		reason:		O p.o O i.v. O n.s.

CHRONIC INFLAMMATORY BOWEL DISEASE (IBD) OF CHILDS AND ADOLESCENTS

ADDITIONAL SHEET

Date of investigation (DD/MM/YYYY): | | | | | | | |

Patient's identification number: | | | - | | | | | |

Indication for surgery

Localization (referring to stenosis as indication for surgery):				
O esophagus	O duodenum	O term. ileum	O rectum	O stoma
O stomach	O prox. ileum, jejunum	O colon	O pouch	

Type of fistula (referring to fistula as indication for surgery):		
O rectovaginal	O enterovesical	O enterocolistic
O enterocutaneous	O enteroenteral	O ending blindly
